# Emerging burden of antimicrobial resistance in Indian hospitals: A review

**DOI:** 10.6026/973206300222664

**Published:** 2026-04-30

**Authors:** Raj Kamal Choudhary, Obaid Ali, Varsha Sinha, Rakhshanda Khatoon

**Affiliations:** 1 Department of Medicine, Jawaharlal Nehru Medical College, Bhagalpur, Bihar, India; 2 Department of Biochemistry, Jawaharlal Nehru Medical College, Bhagalpur, Bihar, India

**Keywords:** Antimicrobial resistance (AMR), stewardship, Bihar

## Abstract

Antimicrobial resistance (AMR) has become a critical global health concern, with a particularly high impact in countries like India. In hospital settings, increasing resistance among pathogens is reducing treatment effectiveness and is associated with greater morbidity, mortality, longer hospital stays and rising healthcare expenditure. Therefore, it is of interest to review (2020-2026) explores the current burden of AMR in India, highlighting resistance trends in common bacterial pathogens and drug-resistant tuberculosis, along with their clinical implications. Key factors driving this problem include inappropriate antibiotic use, easy over-the-counter access, limited diagnostic capacity, inadequate infection control and fragmented surveillance systems. Addressing AMR requires coordinated efforts through antimicrobial stewardship, stronger surveillance, stricter regulation of antibiotic use and improved infection prevention strategies.

## Background:

Antimicrobial resistance (AMR) is now recognized as a major global health threat; accounting for more than 1.2 million deaths annually and projected to cause up to 10 million deaths each year by 2050 if effective interventions are not implemented [1]. The introduction of penicillin by Alexander Fleming revolutionized therapeutic medicine; however, early warnings regarding inappropriate antibiotic use have become a reality, particularly in low- and middle-income countries such as India. India remains a critical epicentre for AMR due to high infectious disease burden, extensive antibiotic utilization and systemic healthcare limitations. Between 2000 and 2015, antibiotic consumption increased by over 100%, exerting substantial selective pressure on microbial populations [3]. Within this context, healthcare facilities in Bihar are increasingly encountering infections caused by multidrug-resistant organisms, consistent with national surveillance trends [4]. Clinically, patients often present with persistent or recurrent infections complicated by inadequate or incomplete prior treatment. Contributing factors include poor adherence, empirical use of broad-spectrum antibiotics and limited access to reliable diagnostic services. Financial constraints further restrict access to newer antimicrobial agents, worsening outcomes [6]. National data continue to highlight the disproportionate burden of AMR in resource-constrained settings [7]. Hospital environments, characterized by high antimicrobial exposure and invasive interventions, provide ideal conditions for the emergence and spread of resistant pathogens. Increasing resistance has been documented in organisms such as Klebsiella, Pseudomonas, Acinetobacter baumannii and Staphylococcus aureus [8].

## Burden and clinical implications of AMR:

AMR continues to escalate as a major public health concern, particularly in low- and middle-income regions [1]. While antimicrobial agents transformed modern medicine, their effectiveness is increasingly compromised due to the rapid emergence of resistant organisms. Consequently, infections that were once easily treatable now require prolonged hospitalization and expensive second- line therapies [8]. At the microbiological level, resistance arises through intrinsic mechanisms or acquired pathways such as genetic mutations and horizontal gene transfer [7]. This has led to the emergence of multidrug-resistant (MDR) and extensively drug-resistant (XDR) pathogens, especially in hospital settings.

## Drivers of AMR in hospital settings in Bihar:

The burden of AMR in tertiary care hospitals is driven by multiple interconnected factors. A key contributor is the irrational use of antibiotics, particularly empirical broad-spectrum therapy without microbiological confirmation [2]. This creates selective pressure favouring resistant strains. In addition, weak antimicrobial stewardship and fragmented surveillance systems hinder evidence-based prescribing. Inadequate infection control practices further promote transmission within healthcare settings. Other contributors include over-the-counter antibiotic access and incomplete treatment courses. Collectively, these factors sustain the persistence and spread of resistance.

## Resistance patterns in Indian hospitals:

Surveillance data from India show a rising prevalence of resistant organisms, particularly in intensive care units. Increasing resistance among gram-negative organisms such as Escherichia coli, Klebsiella pneumoniae and Acinetobacter baumannii has reduced the effectiveness of commonly used antibiotics [4]. The emergence of extended-spectrum beta-lactamase (ESBL) production and carbapenem resistance has further limited treatment options [5]. Tuberculosis remains a major concern, with drug-resistant forms complicating therapy and increasing toxicity risks [3].

## Economic and healthcare burden:

AMR imposes a substantial economic burden, especially in resource-limited settings. Resistant infections are associated with prolonged hospital stays, intensive care needs and higher treatment costs [1]. Financial barriers often lead to delayed or incomplete therapy, worsening outcomes and perpetuating resistance.

## Gaps in surveillance and stewardship:

Despite growing awareness, major gaps persist in AMR control. Surveillance systems remain fragmented, with poor integration across sectors. Variability in implementation of national programs and inconsistent adherence to prescribing guidelines further limit effectiveness [9]. Existing studies are often compartmentalized, restricting comprehensive policy development [5].

## Strategies for mitigation:

Addressing AMR requires a coordinated approach. Strengthening microbiological diagnostics is essential to guide targeted therapy. Antimicrobial stewardship programs can optimize antibiotic use, while stricter regulation of over-the-counter sales is necessary to reduce misuse [2]. Infection prevention measures, including hand hygiene and sterilization, must be rigorously implemented. Improved surveillance systems can generate region-specific data to inform clinical practice [9]. Preserving the effectiveness of existing antibiotics through rational use remains the most practical strategy.

## Broader perspective and future directions:

AMR is not only a microbiological issue but also a systemic healthcare challenge influenced by prescribing behaviour, infrastructure and regulatory gaps. The situation in Bihar reflects broader national trends and highlights the need for integrated interventions. Future strategies should extend beyond hospitals to include environmental control, veterinary regulation and community awareness. A unified, multidisciplinary approach is essential to effectively contain antimicrobial resistance.

## Conclusion:

Antimicrobial resistance represents a growing threat to effective clinical care in Bihar, India. Rising resistance among bacterial pathogens, including multidrug-resistant tuberculosis and gram-negative organisms, has limited therapeutic options and increased healthcare costs. Combating this crisis requires strengthened surveillance, rational prescribing practices, enforcement of antibiotic regulations, improved infection control and sustained public awareness. Institutional commitment and coordinated policy implementation are essential to preserve the effectiveness of antimicrobial therapy and safeguard future generations.

## Advancement to knowledge:

This article highlights evolving resistance patterns among commonly encountered pathogens and underscores gaps between empirical antibiotic practices and local susceptibility trends. By contextualising AMR within routine clinical care, the study offers actionable insights for antimicrobial stewardship programs, supports the need for locally adapted antibiogram and reinforces the urgency of surveillance-driven antibiotic policies. These findings add to the limited granular data from resource-constrained settings and may inform both institutional protocols and broader public health strategies aimed at curbing AMR.
